# Improved collective influence of finding most influential nodes based on disjoint-set reinsertion

**DOI:** 10.1038/s41598-018-32874-5

**Published:** 2018-09-28

**Authors:** Fengkuangtian Zhu

**Affiliations:** Unaffiliated, Shanghai, China

## Abstract

Identifying vital nodes in complex networks is a critical problem in the field of network theory. To this end, the Collective Influence (CI) algorithm has been introduced and shows high efficiency and scalability in searching for the influential nodes in the optimal percolation model. However, the crucial part of the CI algorithm, reinsertion, has not been significantly investigated or improved upon. In this paper, the author improves the CI algorithm and proposes a new algorithm called Collective-Influence-Disjoint-Set-Reinsertion (*CI*_*DR*_) based on disjoint-set reinsertion. Experimental results on 8 datasets with scales of a million nodes and 4 random graph networks demonstrate that the proposed *CI*_*DR*_ algorithm outperforms other algorithms, including Betweenness centrality, Closeness centrality, PageRank centrality, Degree centrality (HDA), Eigenvector centrality, Nonbacktracking centrality and Collective Influence with original reinsertion, in terms of the Robustness metric. Moreover, *CI*_*DR*_ is applied to an international competition on optimal percolation and ultimately ranks in 7th place.

## Introduction

Research on the identification of vital nodes is crucial in computer science, statistical physics and biology applications^1^. Techniques are universally applied in social networks^2–4^, predicting essential proteins^5–7^, quantifying scientific influences^8–10^, detecting financial risks^11–13^, predicting career movements^14,15^ and predicting failures with developer networks^16–18^. Considering its importance for application in many fields, the problem of identifying influencers in a network has attracted substantial attention in network analysis.

Researchers have developed numerous measures to evaluate node importance. The most widely used centrality methods include Betweenness centrality^19^, Closeness centrality^20^, PageRank centrality^21^, Degree centrality (HDA)^22^, Eigenvector centrality^23^, and Nonbacktracking centrality^24^. Betweenness centrality^19^ is defined to represent a node as the number of shortest paths from all vertices to all other paths that pass through that node. Closeness centrality^20^ uses the sum of the length of the shortest paths between the node and all other nodes in a graph as a node’s value. PageRank centrality^21^ was first proposed by Google to rank websites and works by counting the number and quality of links to a page to determine a rough estimate the importance of a website. Degree centrality (HDA)^22^ ranks nodes directly according to the number of connections and recalculates the degree after each removal of the top ranked node. Eigenvector centrality^23^ computes the centrality for a node based on the idea that the importance of a node is recursively related to the importance of the nodes pointing to it. A high eigenvector score means that a node is connected to many nodes who themselves have high scores. However, for some graphs, the Eigenvector centrality will produce an echo chamber effect and localization onto a hub. Nonbacktracking centrality^24^ modified the standard Eigenvector centrality based on the Nonbacktracking matrix to ignore the reflection mechanism on hubs, therein being asymptotically equivalent to Eigenvector centrality for dense networks and avoiding hub localization on sparse networks.

However, for these methods, the node importance is evaluated by regarding a node as an isolated agent in a non-interacting setting. Consequently, these methods are considered as heuristic methods and fail to provide the optimal solution in the general case of finding a single influential node among multiple spreaders^4,25^. To address the issue, a scalable theoretical framework called the Collective Influence (CI) algorithm, which attempts to find the minimal fraction of nodes that can fragment the network in optimal percolation, was recently proposed^4^. If the influencers are removed in a network, the network will face structural collapse, and a giant connected component G of the graph will be 0. The CI would improve and maximize the collective influence of multiple influencers, and the accurate optimization objective is highly adaptable for giving an optimal set of spreaders for various networks. For networks with millions of nodes, such as massive social media and social networks^22^, CI also performs well in processing centrality efficiently.

The implementation of the CI algorithm contains 2 steps. In the first step, CI calculates the value of each node in the network and removes the node with the highest value according to their importance one by one until the giant component is destroyed. Then, in the second step, CI adds back removed nodes and reconstructs the collapsed network, i.e., reinsertion. Reinsertion^4,22^ is the refined post-processing in the CI algorithm and minimizes the giant component G of the graphs for the target G > 0. Although CI has already demonstrated its efficiency in searching for the potential influential nodes in the optimal percolation model, the reinsertion step in CI has rarely been discussed. Until now, the optimal percolation model only addressed the issue of dismantling networks in the first step of CI. The second procedure, reinsertion, is not designed to be optimal, which leads to the fact that the former optimal percolation model is unable to achieve optimal results. Therefore, it is necessary to address this issue by designing a better reinsertion step.

Robustness^26^ is a recently proposed measure for quantifying the performance of methods for ranking nodes. This paper improves the reinsertion method with respect to the Robustness metric to find the most influential nodes in CI, and it proposes a new algorithm named Collective-Influence-Disjoint-Set-Reinsertion (*CI*_*DR*_). *CI*_*DR*_ mainly employs disjoint sets^27^ as the data structure to optimize reinsertion in the CI algorithm and reorder the removed nodes into a new sequence.

The proposed *CI*_*DR*_ method is verified in the International Competition of optimal percolation^28^ and ultimately ranks in 7th place. The competition adopts the Robustness metric as the scoring criteria and provides 4 real networks from different fields, i.e., autonomous system networks, Internet networks, road networks and social networks, and 4 classical artificial networks (8 datasets in total). The node counts of these networks range from 0.4 million to 2 million. Therefore, the competition network benchmark quite representative overall. The results of the experiments indicate that the proposed *CI*_*DR*_ method outperforms the other 7 methods on 8 competition datasets. The methods include Betweenness centrality^19^, Closeness centrality^20^, PageRank centrality^21^, Degree (HDA) centrality^22^, Eigenvector centrality^23^, Nonbacktracking centrality^24^ and Collective Influence with original reinsertion^22^ as comparison algorithms.

A total of 4 extra random graphs in the ER model generated locally are also utilized to verify *CI*_*DR*_. The results on the 4 random graphs show that *CI*_*DR*_ is also better than the other methods listed in the paper, similar to the results on the 8 above competition datasets.

## Results

### Difference between reinsertion in CI and Collective-Influence-Disjoint-Set-Reinsertion (*CI*_*DR*_)

The CI algorithm contains 2 steps: removing nodes and reinsertion. For the removing node step, CI calculates the value of *node*_*i*_ in Formula 1 and removes the node with the highest value.

1 $$C{I}_{l}(i)=({k}_{i}-\mathrm{1)}\sum _{j\in \delta B(i,l)}({k}_{j}-\mathrm{1)}$$

*k*_*i*_ is defined as the degree of *node*_*i*_. *δB*(*i*, *l*) is the frontier of the ball centered on *node*_*i*_ with Radius l, which refers to the shortest path l from frontier nodes to *node*_*i*_^4^. The newly proposed *CI*_*DR*_ method also calculates the value of each node following Formula 1, which is the same as in CI. The difference between CI and *CI*_*DR*_ is that they implement different strategies in the reinsertion step. In CI, the original reinsertion step is invoked^22^ in Algorithm 1 after the networks are broken down into many pieces through the process of removing nodes. An initial collapsed graph *G*_*c*_ is generated after CI removes nodes from the graph G, and then, reinsertion selects the removed nodes to reconstruct the collapsed graph *G*_*c*_.

**Algorithm 1:**
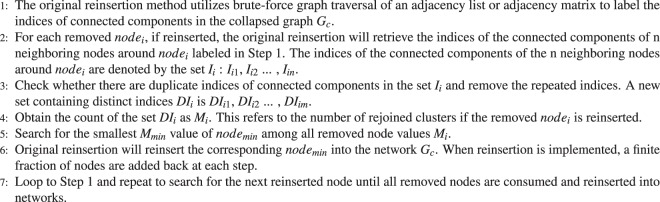
The overall flow of the original reinsertion process in the CI algorithm.

For the reinsertion step in the improved *CI*_*DR*_, 2 main enhancements are proposed in this paper:

- *CI*_*DR*_ implements disjoint sets as the data structure^27^ to store the indices of the connected components during reinsertion for a collapsed graph.
- *CI*_*DR*_ considers the rejoined node count instead of the number of rejoined clusters to decide which node will be reinserted.

In the original reinsertion in Algorithm 1, CI utilizes brute-force graph traversal to label the indices of the connected components in a dismantled network (Step 1). The time cost is high since the labeling operation will be executed multiple times until all removed nodes are reinserted. In particular, when a dismantled network has nearly reached the completion of the reinsertion process and most of the removed nodes are reinserted, the labeling operation must build up from nothing every time. Considering that deciding which node will be reinserted is performed several times in the original reinsertion process, the information of the reinsertion for each iteration can be reserved and prepared for the next round of decisions on which removed node will be reinserted.

The first enhancement of *CI*_*DR*_ implements disjoint sets to optimize the data structure to reduce the computational resource consumption. A disjoint set^27^ is a tree structure, where each node stores a pointer to the parent node. If the parent pointer of a node points to itself, this node is the root of a tree and is the representative index of its cluster. In Algorithm 1, the index information of the connected components in the reconstructed graph is abandoned at the end of each iteration for updating indices. Using the disjoint-set data structure, it is possible to maintain the indices of the connected components for a collapsed network when the dismantled nodes are reinserted into the graphs. The disjoint-set data structure provides 2 nearly constant-time operations. The first operation is called the *Find* operation, which determines which indices of connected components the current nodes stay in. The second operation is the *Union* operation, which merges several clusters into one.

The *Find* operation locates which connected components a node belongs to. The operation can follow the parent node continuously in a tree of a cluster until it finds the root node, which denotes the index of a connected component. The *Find* operation is utilized to replace Step 2 in Algorithm 1 and is capable of retrieving the index set *I*_*i*_ of the connected components of the neighboring nodes around *node*_*i*_.

The *Union* operation merges clusters to which 2 nodes belong into one connected component. This operation uses the *Find* operation to determine the roots of the trees. If the roots of 2 nodes are distinct, the trees are combined by attaching the root of one to the root of the other node. When newly removed nodes are reinserted, the *Union* operation is capable of preserving the index information of the connected components in the iteration when updating indices continuously.

Several optimization methods on disjoint sets, such as *Path Compression* and *Union by Size*, are applied in the implementation to improve the *Find* and *Union* operations^29^. *Path Compression* flattens the structure of the tree by making every node point to the root when the first *Find* operation is invoked on the tree. This will speed up and decrease the complexity of future *Find* operations. *Union by Size* means that the *Union* operation attaches the tree with fewer nodes to the root of the nodes containing more elements. This is also another method for flattening the structure of the tree. The size of the cluster is stored in the root node of a tree, and the new size of the cluster following the *Union* operation is equal to the sum of the sizes of the root nodes of the original trees.

The computational complexity of both the *Find* and *Union* operations is *O*(*inverse*_*oka*(*n*)) when the *Path Compression* and *Union by Size* optimization methods are utilized, where *inverse*_*oka*(*n*) represents the inverse Ackermann function. The inverse Ackermann function contains a value *inverse*_*oka*(*n*) < 5 for any very large value of n that can be written in this physical universe. Therefore, *inverse*_*oka*(*n*) in the *Find* and *Union* operations is optimal and can essentially be regarded as constant time^29,30^. In the experiment analysis section below, the statistical results also show that utilizing the disjoint-set data structure in *CI*_*DR*_ achieves greater efficiency and is faster than the original reinsertion algorithm in CI.

For the second enhancement, *CI*_*DR*_ considers the number of rejoined nodes instead of the number of rejoined clusters when deciding which removed nodes will be reinserted. This improvement is applied in Step 4 in Algorithm 1. The purpose of the modification is to enhance the final Robustness score of the original reinsertion operation in the CI algorithm. The original reinsertion operation adds back the nodes that rejoin the smallest number of clusters. Nevertheless, the method does not consider the smallest node count of the rejoined clusters globally. In contrast to the number of rejoined clusters, the information about the rejoined node counts is more representative for a connected component. Because the first enhancement implements the disjoint-set data structure and because the *Union by Size* optimization is enabled, the node count of each connected component is stored in the root node of the corresponding tree in the disjoint set. The smallest node count of rejoined connected components can be conveniently selected from all candidate nodes.

**Figure 1:**
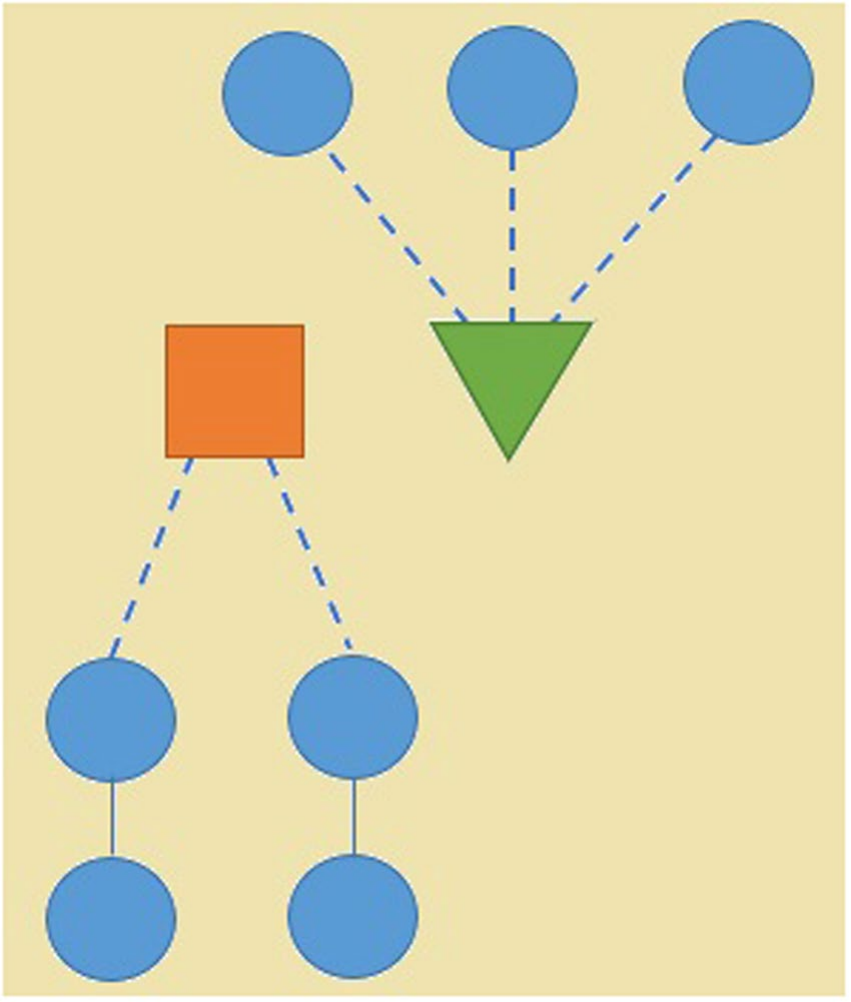
Different choices of candidate reinserted nodes decided by the original reinsertion and *CI*_*DR*_ methods. The original reinsertion method in CI reinserts the square node, and *CI*_*DR*_ reinserts the triangle node.

Figure 1 is an example of different choices of the candidate reinserted nodes decided by the original reinsertion and *CI*_*DR*_ methods. Round nodes have been in the collapsed network, and there are 2 candidate nodes to be reinserted: the square node and the triangle node. If the original reinsertion method in CI is applied, it will reinsert the square node because the number of rejoined clusters is 2; fewer than 3 clusters are reinserted by the triangle node. If *CI*_*DR*_ is exploited, it will reinsert the triangle node because there are 3 rejoined nodes, and fewer than 4 rejoined nodes are reinserted by the square nodes. *CI*_*DR*_ more strongly considers the global impact of the rejoined nodes on the collapsed network compared with the original reinsertion process. In conclusion, the *CI*_*DR*_ algorithm with the first and second enhancements for the reinsertion process is shown in Algorithm 2.

**Algorithm 2:**
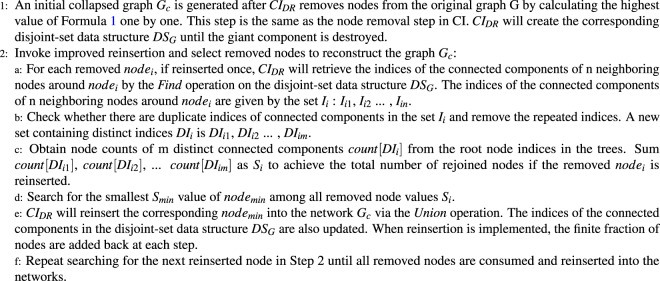
Collective-Influence-Disjoint-Set-Reinsertion (C*I*_*DR*_).

### Experiments and comparison of different methods on 8 datasets provide by DataCastle Master Competition

In this subsection, several centrality methods are verified on 8 datasets provided by the DataCastle Master Competition^28^. The task of the competition is a generic challenge identifying vital nodes in networks that are important for sustaining connectivity. The competition provides 4 real networks from different fields, i.e., autonomous system networks, Internet networks, road networks and social networks, and 4 classical artificial networks (for a total of 8 datasets). These networks each include 0.4 million to 2 million nodes, and all networks are considered undirected networks. Table 1 reflects the network name and corresponding number of nodes.

**Table 1:** The 8 network datasets in the DataCastle Master Competition.

Network	model1	model2	model3	model4	real1	real2	real3	real4
Number of nodes	1039722	1083568	997663	1001733	1694616	1957027	426485	855802

Robustness^26^ is utilized as the scoring criterion in the competition. It is introduced to quantify the performance of the methods for ranking nodes. For the calculation of the Robustness score, refer to Formula 2.

2 $$R=\frac{1}{N}\sum _{i=1}^{N}\delta (\frac{i}{n})$$

The parameter p is defined as the proportion of removed nodes. *δ* is the size of the giant component of the remaining networks in proportion after removing a proportion p of the nodes. The *δ*-p curve can be derived from plotting p on the x-axis and *δ* on the y-axis. The Robustness is defined as the area under the *δ*-p curve. $\delta (\frac{i}{n})$ is the size of the giant component after removing $p=\frac{i}{n}$ of the nodes from a network^28^.

Generally, the goal of an algorithm that finds the most influential nodes is to give a ranked list of nodes according to their importance, where the top-ranked nodes will have greater importance. Nodes can be removed from a network according to the ranking list. The removal operation breaks down the network into many disconnected pieces. If the size of the giant component is calculated after the removal of each node, the ratio of the giant component will ultimately go to 0. Therefore, a better algorithm for ranking nodes will dismantle networks sooner and produce better Robustness scores.

**Figure 2:**
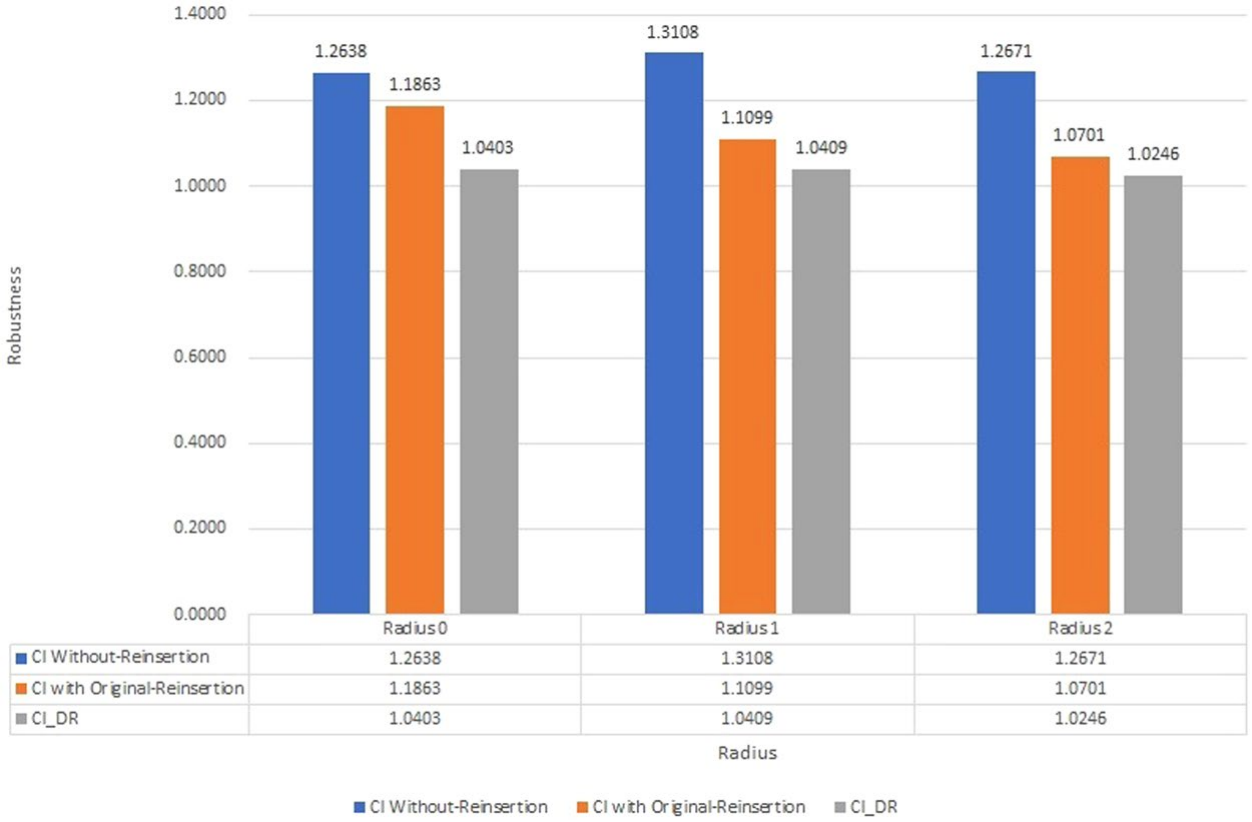
Total Robustness score of CI Without Reinsertion, with Original Reinsertion and *CI*_*DR*_ on 8 competition datasets for radii of 0, 1, and 2.

The Robustness under CI without Reinsertion, with Original Reinsertion and *CI*_*DR*_ on 8 competition datasets is presented in Table 2 and Fig. 2. CI without Reinsertion refers to the case in which CI only invokes the process of removing nodes and does not reinsert nodes into networks. CI with Original Reinsertion refers to the case with the node removal step and original reinsertion in Algorithm 1. For various similar CI algorithms, the radius is the required input parameter for the node removal step. A larger radius will optimize removing steps and produce a smaller set of minimal influential nodes when dismantling networks. As a trade-off, the step of removing nodes will cost more computing resources and take longer. Meanwhile, a larger radius will speed up reinsertion because fewer dismantled nodes are reinserted. The result of using different input radii of 0, 1, and 2 for the various CI methods is also shown in Table 2 and Fig. 2.

**Table 2:** Robustness score of CI without Reinsertion, with Original Reinsertion and *CI*_*DR*_ on 8 competition datasets for radii of 0, 1, and 2.

Radius 0	model 1	model 2	model 3	model 4	real 1	real 2	real 3	real 4	total
CI Without Reinsertion	0.2279	0.1914	0.3794	0.1446	0.0493	0.0689	0.1102	0.0922	1.2638
CI with Original Reinsertion	0.2130	0.1783	0.3488	0.1304	0.0459	0.0918	0.1030	0.0751	1.1863
*CI*_*DR*_	0.2100	0.1744	0.3603	0.1175	0.0315	0.0069	0.0978	0.0417	1.0403
**Radius 1**	**model 1**	**model 2**	**model 3**	**model 4**	**real 1**	**real 2**	**real 3**	**real 4**	**total**
CI Without Reinsertion	0.2253	0.1878	0.3809	0.1434	0.0523	0.1069	0.1118	0.1024	1.3108
CI with Original Reinsertion	0.2079	0.1729	0.3459	0.1258	0.0407	0.0545	0.0969	0.0654	1.1099
*CI*_*DR*_	0.2104	0.1743	0.3656	0.1152	0.0318	0.0046	0.0968	0.0421	1.0409
**Radius 2**	**model 1**	**model 2**	**model 3**	**model 4**	**real 1**	**real 2**	**real 3**	**real 4**	**total**
CI Without Reinsertion	0.2239	0.1867	0.3777	0.1317	0.0482	0.0941	0.1085	0.0962	1.2671
CI with Original Reinsertion	0.2083	0.1732	0.3446	0.1189	0.0387	0.0417	0.0955	0.0492	1.0701
*CI*_*DR*_	0.2107	0.1739	0.3585	0.1148	0.0303	0.0039	0.0954	0.0370	1.0246

Table 2 and Fig. 2 show that the total Robustness (the lower, the better) of *CI*_*DR*_ on the 8 datasets is better than in the cases without Reinsertion and with Original Reinsertion. This is because in the reinsertion process, the rejoined node count is more representative in vital nodes than the number of rejoined clusters in the Robustness metric. For a radius of 0, the total Robustness score under CI with Original Reinsertion of 1.1863 decreases by 12% to 1.0403 for *CI*_*DR*_. For a radius of 1, the total Robustness score of 1.1099 for CI decreases by 6% to 1.0409 for *CI*_*DR*_. For a radius of 2, the total Robustness score of 1.0701 decreases 4% to 1.0246 for *CI*_*DR*_. For each individual dataset, *CI*_*DR*_ performs better than CI in terms of Robustness in 7 of the 8 networks when a radius of 0 is used in CI and *CI*_*DR*_. *CI*_*DR*_ ranks second to CI only in the model 3 network. When a radius of 1 and a radius of 2 are adopted in CI and *CI*_*DR*_, *CI*_*DR*_ obtains a better score than CI on 5 of the 8 networks. *CI*_*DR*_ ranks second behind CI in the model 1, model 2 and model 3 networks and obtains nearly the same and best result.

For different radii as input parameters, the total Robustness values in *CI*_*DR*_ are all better than those with Original Reinsertion. Even for the case when the radius is 0 and the process of removing nodes degenerates into that of Degree centrality (HDA)^22^, *CI*_*DR*_ is capable of achieving a considerably better score of 1.0403 compared with the previous best result of 1.0701 under Original Reinsertion with a radius of 2. For the smaller radius, *CI*_*DR*_ exploits potential performance increases in terms of the Robustness metric, in contrast to Original Reinsertion. Therefore, there is no need to set a higher radius, which increases the complexity of removing nodes in CI. A lower radius is able to achieve nearly the same results in *CI*_*DR*_.

**Figure 3:**
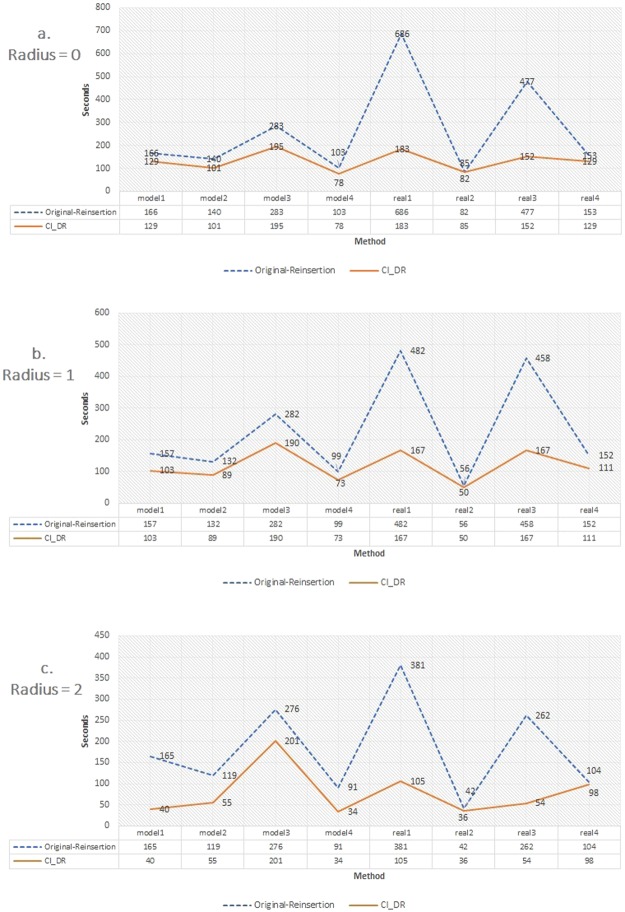
Time consumption (seconds) of Original Reinsertion and *CI*_*DR*_ excluding the step of removing nodes on 8 competition datasets. (**a**) Radius = 0. (**b**) Radius = 1. (**c**) Radius = 2.

In Table 3 and Fig. 3, the time consumption of Original Reinsertion and *CI*_*DR*_ excluding removing nodes on 8 competition datasets is presented. The datasets are verified on the same machine with a 4-core CPU (Intel Xeon E5-2667v4 Broadwell 3.2 GHz) with 8 GB of memory concurrently. For most cases with different radii, the statistics show that *CI*_*DR*_ is better in terms of speed than Original Reinsertion, excluding the node removal steps, in CI. For the real 3 dataset with a radius of 2, the time consumption can be reduced 79.3% from 262 s to 54 s. The statistics evidence that implementing the disjoint-set data structure in *CI*_*DR*_ is more efficient than the Original Reinsertion algorithm.

**Table 3:** Time consumption of Original Reinsertion and *CI*_*DR*_ excluding the step of removing nodes on 8 competition datasets.

Radius 0	model	model 2	model 3	model 4	real 1	real 2	real 3	real 4
Original Reinsertion	166s	140s	283s	103s	686s	82s	477s	153s
*CI*_*DR*_	129s	101s	195s	78s	183s	85s	152s	129s
**Radius 1**	**model 1**	**model 2**	**model 3**	**model 4**	**real 1**	**real 2**	**real 3**	**real 4**
Original Reinsertion	157s	132s	282s	99s	482s	56s	458s	152s
*CI*_*DR*_	103s	89s	190s	73s	167s	50s	167s	111s
**Radius 2**	**model 1**	**model 2**	**model 3**	**model 4**	**real 1**	**real 2**	**real 3**	**real4**
Original Reinsertion	165s	119s	276s	91s	381s	42s	262s	104s
*CI*_*DR*_	40s	55s	201s	34s	105s	36s	54s	98s

**Figure 4:**
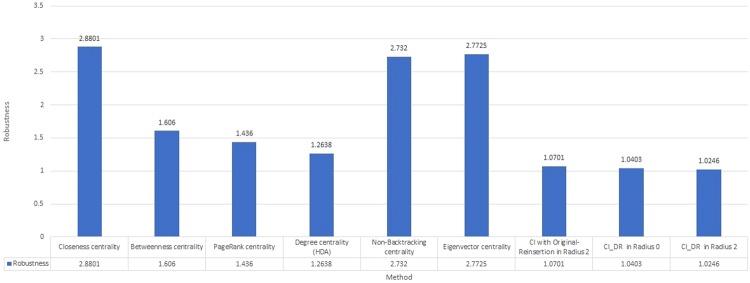
Total Robustness of different heuristic algorithms, including Betweenness centrality, Closeness centrality, PageRank centrality, Degree centrality (HDA), Eigenvector centrality and Nonbacktracking centrality, on 8 competition datasets

Heuristic algorithms, including Betweenness centrality^19^, Closeness centrality^20^, PageRank centrality^21^, Degree centrality (HDA)^22^, Eigenvector centrality^23^ and Nonbacktracking centrality^24^, are verified on these datasets as competitors in Table 4 and Fig. 4. The Robustness values of these heuristic algorithms are all worse than CI and *CI*_*DR*_ for the 8 competition networks. The worst value of *Closeness centrality* is only 2.88.

**Table 4:** Robustness of different heuristic algorithms, including Betweenness centrality, Closeness centrality, PageRank centrality, Degree centrality (HDA), Eigenvector centrality and Nonbacktracking centrality, on 8 competition datasets.

	model 1	model 2	model 3	model 4	real 1	real 2	real 3	real 4	Total
Betweenness centrality	0.3125	0.2678	0.4484	0.1952	0.1101	0.0064	0.1582	0.1076	1.6060
Closeness centrality	0.4140	0.3765	0.4624	0.3717	0.2495	0.4454	0.2738	0.2868	2.8801
PageRank centrality	0.2435	0.2043	0.4267	0.1469	0.0522	0.1364	0.1430	0.0832	1.436
Degree centrality (HDA)	0.2279	0.1914	0.3794	0.1446	0.0493	0.0689	0.1102	0.0922	1.2638
Eigenvector centrality	0.4228	0.4163	0.4624	0.3938	0.2718	0.2222	0.2892	0.2939	2.7725
Nonbacktracking centrality	0.4142	0.3850	0.4624	0.3757	0.2715	0.2443	0.2885	0.2905	2.7320

The recently proposed Nonbacktracking centrality^24^ is also verified on these 8 competition datasets. Nonbacktracking centrality was introduced by Newman *et al*. and modified from the standard Eigenvector centrality based on the Hashimoto or Nonbacktracking matrix^31–33^. Nonbacktracking centrality is very similar to Eigenvector centrality, where the main improvement is to ignore the echo chamber effect producing localization on a hub. This is asymptotically equivalent to Eigenvector centrality for dense networks and avoids the hub localization on sparse networks introduced by Eigenvector centrality. Therefore, the performance of Nonbacktracking centrality in dense networks will be highly similar to Eigenvector centrality. From Table 4 and Fig. 4, the statistics show that corresponding scores of 2.7725 and 2.7320 for Eigenvector centrality and NonBacktracking centrality, respectively, are quite similar. Both Nonbacktracking centrality and Eigenvector centrality are not superior to CI and the proposed *CI*_*DR*_ in terms of Robustness for the 8 competition datasets.

### Experiments and comparison of different methods on 4 randomly generated graphs

**Figure 5:**
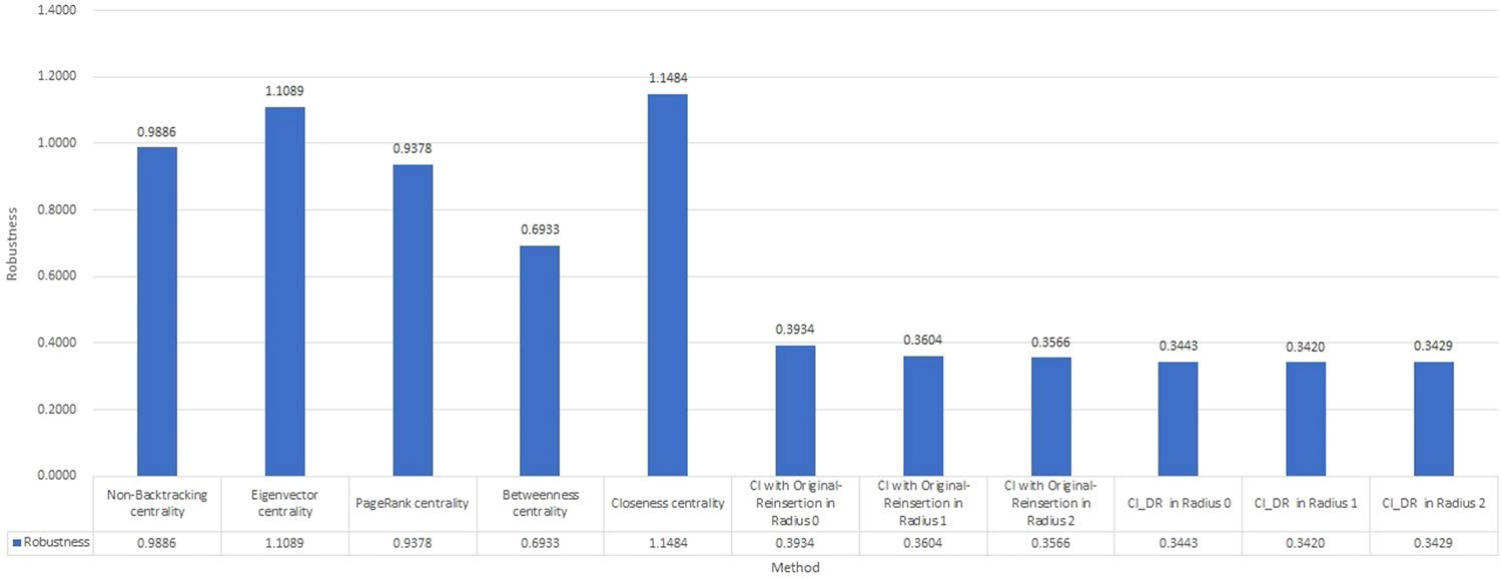
Total Robustness value of CI with Original Reinsertion, *CI*_*DR*_ and other heuristic algorithms, including Betweenness centrality, Closeness centrality, PageRank centrality, Eigenvector centrality and Nonbacktracking centrality, on 4 random graphs.

In addition to the 8 above-mentioned competition datasets, 4 random graph networks in the ER model^34^ are also adopted as additional test cases. Table 5 shows the information about the number of nodes and mean degree for each graph. The Robustness values under CI with Original Reinsertion, *CI*_*DR*_ and other heuristic algorithms, including Betweenness centrality, Closeness centrality, PageRank centrality, Eigenvector centrality and Nonbacktracking centrality, are presented in Table 6 and Fig. 5 for 4 random graphs.

**Table 5:** 4 network datasets for randomly generated graphs in the ER model.

Network	Random 0	Random 1	Random 2	Random 3
Number of nodes	10^5^	10^5^	10^6^	10^6^
Mean degree	2	3	2	3

**Table 6:** Robustness value of CI with Original Reinsertion, *CI*_*DR*_ and other heuristic algorithms, including Betweenness centrality, Closeness centrality, PageRank centrality, Eigenvector centrality and Nonbacktracking centrality, on 4 random graphs.

Network	Random 0	Random 1	Random 2	Random 3	Total
Betweenness centrality	0.1169	0.2232	0.1209	0.2323	0.6933
Closeness centrality	0.2687	0.3045	0.2685	0.3068	1.1484
PageRank centrality	0.1740	0.2962	0.1736	0.2941	0.9378
Eigenvector centrality	0.2482	0.3036	0.2501	0.3070	1.1089
Nonbacktracking centrality	0.2001	0.2947	0.1971	0.2967	0.9886
CI with Original Reinsertion (Radius = 0)	0.0654	0.1326	0.0628	0.1326	0.3934
CI with Original Reinsertion (Radius = 1)	0.0560	0.1252	0.0542	0.1250	0.3604
CI with Original Reinsertion (Radius = 2)	0.0548	0.1248	0.0523	0.1246	0.3566
*CI*_*DR*_ (Radius = 0)	0.0492	0.1234	0.0480	0.1237	**0.3443**
*CI*_*DR*_ (Radius = 1)	0.0489	0.1218	0.0490	0.1224	**0.3420**
*CI*_*DR*_ (Radius = 2)	0.0484	0.1214	0.0480	0.1251	**0.3429**

The total Robustness of *CI*_*DR*_ is 0.3443, 0.3420 and 0.3429 for radii of 0, 1, and 2 on 4 randomly generated graphs, which all outperform the other listed centrality methods. For each individual dataset, the Robustness of *CI*_*DR*_ also ranks 1st out of all applied methods. For a radius of 0, *CI*_*DR*_ performs better in terms of Robustness than CI with Original Reinsertion for a radius of 2. The same result as that on the 8 above-mentioned competition datasets that a lower radius under *CI*_*DR*_ outperforms a higher radius under CI with Original Reinsertion is found.

The NonBacktracking centrality achieves a score of 0.9886, which is slightly better than the score of 1.1089 of the Eigenvector centrality; this is because the former method is based on the latter method. However, a score of 0.9886 is unable to compete with CI and *CI*_*DR*_, similar to the results on the 8 above competition datasets.

## Discussion

For 8 competition datasets and 4 local randomly generated graphs under the ER model, the best overall result from the previous algorithms is CI with Original Reinsertion for a radius of 2. After the newly proposed algorithm *CI*_*DR*_ is applied, even *CI*_*DR*_ employing a radius of 0 (degenerate to HDA) is capable of achieving a better result. This indicator shows that the proposed disjoint-set reinsertion in *CI*_*DR*_ is able to achieve better Robustness compared to Original Reinsertion. The recently proposed Nonbacktracking centrality and the other above-mentioned algorithms are also unable to outperform *CI*_*DR*_ in terms of Robustness.

CI with Original Reinsertion uses the number of rejoined clusters to decide which node will be reinserted. On the other hand, *CI*_*DR*_ considers the rejoined node count in the second proposed enhancement. Nevertheless, CI with Original Reinsertion and *CI*_*DR*_ implement different methods; both methods attempt to obtain a score that is capable of representing *node*_*i*_. Therefore, a reinsertion framework derived from *CI*_*DR*_ can be extended to a more general model. The Generic Disjoint-set Reinsertion Framework (GDRF) is proposed in Algorithm 3 as a general method for describing the process of disjoint-set reinsertion.

**Algorithm 3:**
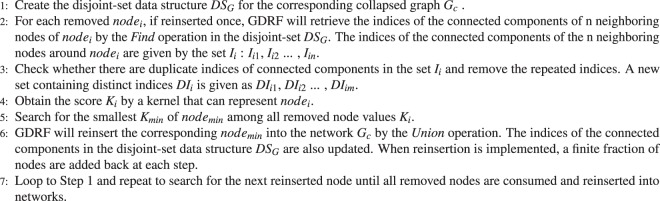
Algorithm of reinsertion with kernel in a more generic framework: Generic Disjoint-set Reinsertion Framework (GDRF).

Step 4 in Algorithm 3 retrieves the score *K*_*i*_ by a specific kernel indicating *node*_*i*_, and the other steps in Algorithm 3 are the same as in the reinsertion in the *CI*_*DR*_ Algorithm 2. GDRF employing the *number Of Clusters* kernel (Algorithm 4) and the *number Of Nodes* kernel (Algorithm 5) is able to achieve the same Robustness score as CI with Original Reinsertion and *CI*_*DR*_, respectively.

**Algorithm 4:**
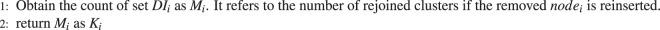
CI with Original Reinsertion: number of Clusters kernel used to obtain the score *K*_*i*_ representing *node*_*i*_.

**Algorithm 5:**
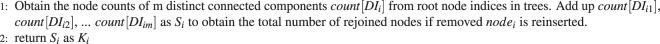
*CI*_*DR*_: *numberOfNodes* kernel used to obtain the score *K*_*i*_ representing *node*_*i*_.

Since the reinsertion of independent post-processing can be combined with any network dismantling method to obtain an improved Robustness score, the greater potential of GDRF can be investigated. For the task of finding the most influential nodes in complex networks, the *number Of Clusters* kernel and the *number Of Nodes* kernel are implemented to reinsert the nodes with less importance in priority.

In future work, more kernels can be studied to investigate whether GDRF can be applied to other issues. For instance, reconstructing damaged networks is also a widely studied field, and various repair strategies have been presented to repair collapsed networks^35–39^. A new kernel for GDRF, which would be designed to reinsert the nodes with greater importance in priority, can be developed for the task of recovering attacked networks as soon as possible. The *Recover Nodes* kernel in Algorithm 6 combined with GDRF is an example of selecting the nodes with greater importance in priority. *S*_*i*_ represents the total number of rejoined clusters if the removed *node*_*i*_ is reinserted. A larger *S*_*i*_ means that recovering *node*_*i*_ would connect and repair more connected components in the reinsertion process. Compared with the random reinsertion of removed nodes, the *recover Nodes* kernel tends to reinsert nodes combining with more connected components, which means that recovering a network to certain giant components G > 0 would need fewer reinserted nodes. Since GDRF will search for the minimum *K*_*min*_ value of *node*_*min*_ among all removed node values *K*_*i*_, Algorithm 6 would return the reciprocal of *S*_*i*_ as *K*_*i*_, and *node*_*i*_ with larger values of *S*_*i*_ would be reinserted in priority.

**Algorithm 6:**
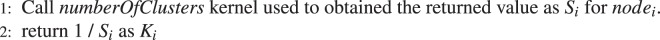
Recovering attacked network: *recoverNodes* kernel used to obtain the score *K*_*i*_ representing *node*_*i*_.

The *RecoverNodes* kernel is only suitable when the nodes in a network, instead of the edges, are attacked. The *RecoverNodes* kernel will also not modify the original network topology after the recovery process. Because this paper mainly focuses on the influential nodes and not repairing attacked networks, additional research on the performance of the *recoverNodes* kernel compared with previous algorithms can be conducted in future studies.

## Methods

As mentioned above, *CI*_*DR*_ calculates the value of each node of a network using Formula 1 and removes the nodes with the highest value. In particular, if the radius is set to 0, the step of removing nodes in CI and *CI*_*DR*_ will degenerate to the High Degree Adaptive (HDA) algorithm. The concept of HDA was proposed^4^ as a better strategy and is slightly different from the original Degree centrality method. The degree of the remaining nodes in adaptive HDA is recomputed after each node removal.

To verify CI and *CI*_*DR*_ on the datasets, 2 implementations of the algorithm are utilized: CI_HEAP^40^ and ComplexCi^41^. CI_HEAP was provided by the original paper written in the C language and generates the statistics of the Original Reinsertion method. ComplexCi is newly developed as a C++ implementation and produces the statistics of *CI*_*DR*_. CI_HEAP and ComplexCi share the same parameters as follows:

- The start points of the reinsertion in CI_HEAP and ComplexCi are the same. Both start to reinsert a node when the size of the giant component collapses to 1% of the whole network.
- The finite fractions of nodes at each reinserted step in CI_HEAP and ComplexCi are the same, and both methods reinsert 0.1% at each step.
- The intervals of the computing component in CI_HEAP and ComplexCi are the same. To determine whether 1% of the giant component has been reached, CI_HEAP and ComplexCi both need to compute the size of the giant component periodically. The interval parameter is 1%, which means that they will calculate the giant component after the CI algorithm removes 1% of the network nodes.
  There are several differences between CI_HEAP and ComplexCi when implementing their algorithms as follows.
- CI_HEAP uses the Original Reinsertion method, and ComplexCi uses *CI*_*DR*_.
- Compared with the initial proposed CI^4^, CI_HEAP enhances the algorithm by utilizing the max-heap data structure^22^ for very efficiently processing the CI values. The computational complexity of CI is O(Nlog N) when removing nodes one by one, made possible through an appropriate data structure for processing CI. The ComplexCi application uses a red-black tree with the STL (Standard Template Library) container SET as a different data structure to store and update the CI values. In the field of C++ programming, the SET and MAP containers in STL are usually implemented as red-black trees, which are a type of self-balancing binary search tree. The average computational complexity of a red-black tree in searching, inserting and deleting nodes is O(log N). Although the red-black tree does not outperform the performance of deleting and updating, in contrast to max-heap, red-black tree is still able to achieve an overall computational complexity of O(Nlog N).
- As mentioned in algorithm 2, when the reinsertion is implemented in the experimental section, the top 0.1% of qualified nodes are added back at each step. For instance, if we have 2000 removed nodes, reinsertion will add back 0.1% * 2000 = 20 nodes at each step until all nodes are once again in the network. Hence, we need to choose 20 nodes with the minimal value *S*_*min*_ out of the total of 2000 candidates at each reinsertion. Original Reinsertion implements a direct quick sort algorithm of O(Nlog N) to sort all nodes and obtain the top nodes. In *CI*_*DR*_, *Introselect algorithm*^42^ is used to select the top N qualified nodes without a sort algorithm, therein simply being of O(N). We do not need to know the order of the *S*_*min*_ array using full sort; we simply need to know the top N qualified nodes.

For Betweenness centrality^19^, Closeness centrality^20^ and PageRank centrality^21^, a complex network python library GraphTools^43^ is utilized to obtain the statistics. For Eigenvector centrality and Nonbacktracking centrality, the python tool NetworkX^44^ is implemented to generate the statistics. To obtain the Nonbacktracking centrality of a network, if the leading eigenvector of its Nonbacktracking matrix B is computed directly according to the definition, the computational complexity will be high. In practice, a faster computation can be executed by utilizing the so-called Ihara (or Ihara-Bass) determinant formula^31,45,46^. It can be shown that the centralities on the Nonbacktracking matrix are equal to the first n elements of the leading eigenvector of the 2N * 2N matrix in Formula 3:

3 $$M=(\begin{array}{cc}A & I-D\\ I & 0\end{array})$$

where *A* is the adjacency matrix, *I* is the identity matrix, and *D* is the diagonal matrix, with the degrees of the vertices along the diagonal^24^.

As mentioned above, the 8 competition datasets in the paper were obtained from the DataCastle Master Competition^28^, therein providing 4 real networks and 4 classical artificial networks. The 4 extra randomly generated graphs under the ER model are generated locally by the python utility NetworkX^44^.

For the calculation of the Robustness score, the code used in this paper is implemented from the DataCastle Master Competition and can be found at the official DataCastle website^47^.
