# Publisher Correction: Improved collective influence of finding most influential nodes based on disjoint-set reinsertion

**DOI:** 10.1038/s41598-018-35525-x

**Published:** 2018-11-20

**Authors:** Fengkuangtian Zhu

**Affiliations:** Unaffiliated, Shanghai, China

Correction to: *Scientific Reports* 10.1038/s41598-018-32874-5, published online 28 September 2018

The original version of this Article contained typographical errors in the Abstract.

“To this end, the Collective. Influence (CI) algorithm has been introduced and shows high efficiency and scalability in searching for the influential nodes in. the optimal percolation model. However, the crucial part of the CI algorithm, reinsertion, has not been significantly investigated. or improved upon.”

now reads:

“To this end, the Collective Influence (CI) algorithm has been introduced and shows high efficiency and scalability in searching for the influential nodes in the optimal percolation model. However, the crucial part of the CI algorithm, reinsertion, has not been significantly investigated or improved upon.”

This has now been corrected in the PDF and HTML versions of the Article.

